# Integrative Mendelian randomization reveals the soluble receptor for advanced glycation end products as protective in relation to rheumatoid arthritis

**DOI:** 10.1038/s41598-023-35098-4

**Published:** 2023-05-17

**Authors:** Gha Young Lee, Chen Yao, Shih-Jen Hwang, Jiantao Ma, Roby Joehanes, Dong Heon Lee, R. Curtis Ellison, Lynn L. Moore, Chunyu Liu, Daniel Levy

**Affiliations:** 1 Population Sciences Branch, Division of Intramural Research, National Heart, Lung, and Blood Institute, National Institutes of Health, Bethesda, MD USA; 2 Framingham Heart Study, 73 Mt. Wayte Avenue, Framingham, MA 01702 USA; 3 School of Nutrition Science and Policy, Tufts University, Boston, USA; 4 Boston University School of Medicine, Boston, MA USA; 5 School of Public Health, Boston University, Boston, MA USA

**Keywords:** Cardiovascular diseases, Rheumatoid arthritis, Data integration, Genome informatics, Proteome informatics, Computational models

## Abstract

Rheumatoid arthritis (RA) is a risk factor for atherosclerotic cardiovascular diseases (CVD). Given the critical roles of the immune system and inflammatory signals in the pathogenesis of CVD, we hypothesized that interrogation of CVD-related proteins using integrative genomics might provide new insights into the pathophysiology of RA. We utilized two-sample Mendelian randomization (MR) for causal inference between circulating protein levels and RA by incorporating genetic variants, followed by colocalization to characterize the causal associations. Genetic variants from three sources were obtained: those associated with 71 CVD-related proteins measured in nearly 7000 Framingham Heart Study participants, a published genome-wide association study (GWAS) of RA (19 234 cases, 61 565 controls), and GWAS of rheumatoid factor (RF) levels from the UK Biobank (*n* = 30 565). We identified the soluble receptor for advanced glycation end products (sRAGE), a critical inflammatory pathway protein, as putatively causal and protective for both RA (odds ratio per 1-standard deviation increment in inverse-rank normalized sRAGE level = 0.364; 95% confidence interval 0.342–0.385; *P* = 6.40 × 10^–241^) and RF levels (β [change in RF level per sRAGE increment] = − 1.318; SE = 0.434; *P* = 0.002). Using an integrative genomic approach, we highlight the *AGER*/RAGE axis as a putatively causal and promising therapeutic target for RA.

## Introduction

Rheumatoid arthritis (RA) is one of the most common chronic autoimmune diseases with a worldwide prevalence of 0.5–1% in adults^1^. Risk factors for RA include a strong genetic component^2^, prompting large-scale genome-wide association studies (GWAS) that have revealed more than 100 RA-associated genetic loci^2^.

RA is also a risk factor for cardiovascular disease (CVD) and multiple studies demonstrate a 1.5 to 2-fold risk of coronary artery disease in RA patients^3–6^. Currently hypothesized mechanisms for the predisposition to CVD among RA patients include shared genetic and environmental risk factors and dysregulation of inflammation and immune function^4,7^. Indeed, it was recently shown that baseline inflammatory markers such as erythrocyte sedimentation rate and C-reactive protein levels were associated with higher heart failure risk at 5- and 10-yr follow up^8^. There also have been recent early murine models and cross-sectional studies showing the link of IL-6 trans-signalling in the progression of atherosclerosis in RA^9,10^, as well as the role of annexin A1 in interrupting the progression of cardiomyopathy in arthritis models^11^. The exact immune-mediated and inflammatory mechanisms linking RA and CVD, however, are unknown and warrant elucidation.

## Materials and methods

We analysed causal relations of 71 CVD-associated proteins to RA using protein quantitative trait loci (pQTL) from GWAS of plasma protein levels in 6,861 Framingham Heart Study (FHS) participants^12^ in conjunction with large-scale GWAS of RA^13^ and circulating rheumatoid factor (RF) levels^14^, which reflects anti-IgG immunoglobulins present in 80–90% of patients with RA^15^.

To assess the potential causal association between CVD-related proteins and RA, we employed Mendelian randomization^16^ (MR), a statistical approach to infer causality of an exposure for an outcome by mimicking randomized control trials using genetic variants as instrumental variables (IVs; Fig. S1), and colocalization, a Bayesian approach to assess shared genetic signals for two traits^17^. We applied two-sample MR^16^ to identify proteins causally associated with RA and RF and assessed the probability that the signals from MR are due to shared genetic variants^18,19^. While colocalization is not a test of causal inference between the exposure and the outcome, it identifies shared genetic variants, and when carried out in conjunction with Mendelian randomization can both provide additional insight into the mechanism of the causal association and reduce the probability of horizontal pleiotropy^19,20^. We further hypothesized that this integrative genomics approach might reveal CVD-related proteins that are causally linked to RA, thereby highlighting promising targets for the treatment of RA.

### Study design

**Figure 1:**
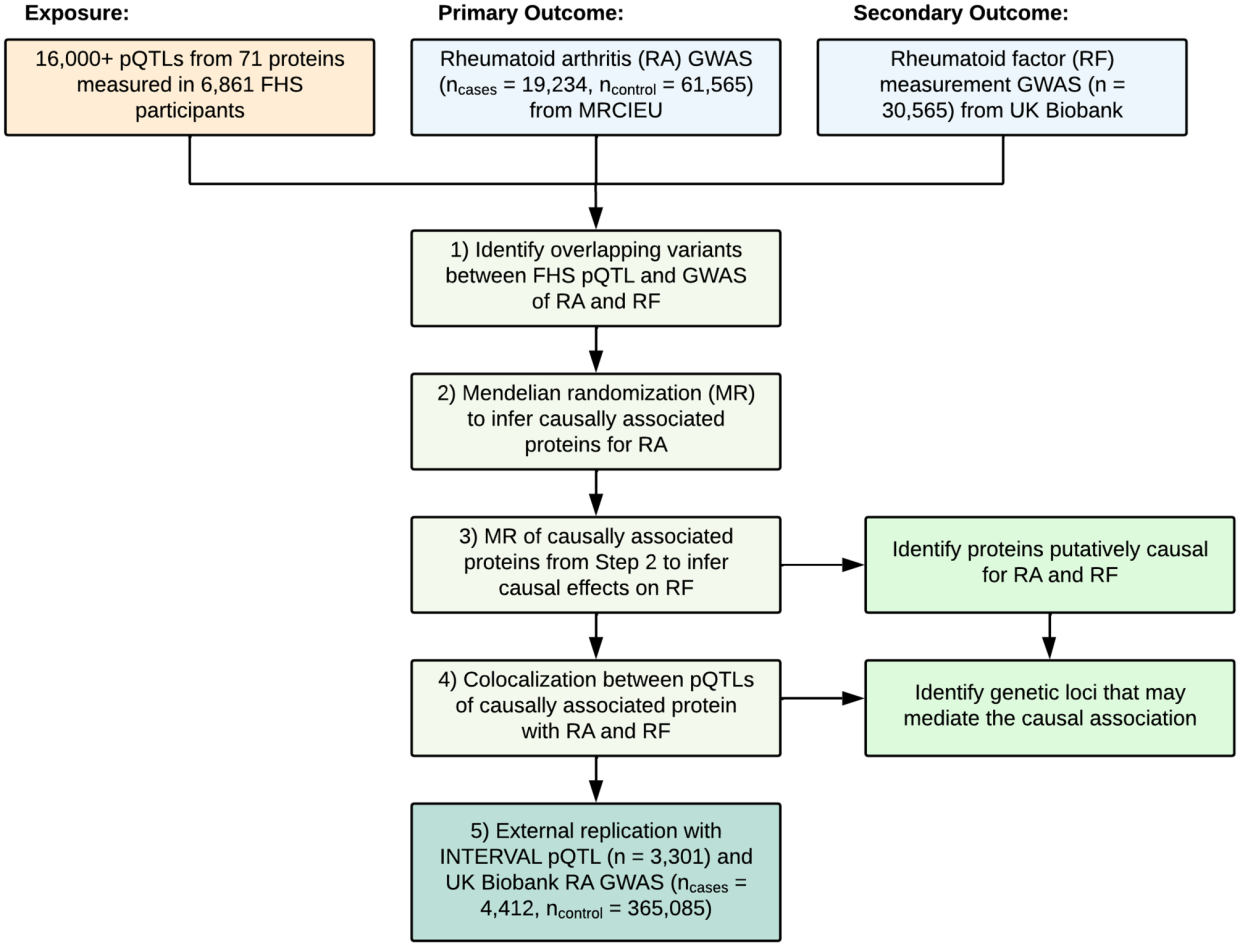
Study design. Flowchart of the study design. The study consisted of four steps: i. Identify pQTL variants overlapping with genetic variants for RA from GWAS, ii. and iii. Mendelian randomization analyses of the primary and secondary traits, iv. Colocalization analysis using the pQTL and GWAS, and v. Replication utilizing external pQTL and GWAS. The GWAS for RA was obtained via MRCIEU^13,16^ and the GWAS for RF^14^ was obtained via the UK Biobank. v. External replication was then achieved with the INTERVAL pQTL^21^ and the GWAS for RA^22^ obtained via the UK Biobank.

The study consisted of five steps (Fig. 1). First, from over 16,000 pQTL variants identified from GWAS of 71 CVD-related proteins measured in 6861 FHS participants^12^, we characterized pQTL variants that coincided with genetic variants from GWAS of RA^13,16^. Second, using *cis-*pQTL variants (i.e. residing within 1 Mb of the protein-coding gene) as IVs, we conducted MR testing to infer causal effects of proteins on RA (Fig. S1). Third, any causal protein from RA MR analysis was subject to MR analysis investigating its causal effect on RF levels. Fourth, colocalization analysis was performed on the putatively causal protein with RA to tease out potential loci that modulate the causal association. Fifth, MR analysis for a causal protein from the primary MR analysis was repeated with external replication with pQTLs from the INTERVAL study^21^ and a smaller GWAS of RA from the UK Biobank^22^.

The 71 plasma proteins were selected based on their relation to CVD as described previously^12^. Protein levels in FHS participants were measured using Luminex bead-based assays (Luminex, Inc, Austin, TX)^23^. Genotyping of participants was performed using Affymetrix genotyping arrays (Affymetrix, Inc, Santa Clara, CA) as well as the Illumina Exome Chip array (Illumina, Inc. San Diego, CA). GWAS of inverse-rank normalized protein levels was performed in R and SAS using genotype dosages based on 1000 Genomes Project imputation (Affymetrix genotypes) or observed genotypes (Exome Chip) in linear mixed-effects models^12^.

Our primary analysis used the UK Medical Research Council Integrative Epidemiology Unit’s (MRCIEU) summary statistics for the trans-ethnic GWAS of RA (19 234 cases, 61 565 controls) by Okada et al.^13,16^. All RA cases fulfilled diagnostic criteria of the American College of Rheumatology or were diagnosed by a rheumatologist^13^. Secondary analysis was conducted on the UK Biobank GWAS of circulating RF levels (*n* = 30 565)^14^. The protocols for measuring serum RF and genotyping are described elsewhere^14^. Replication analyses were conducted with the INTERVAL pQTLs (*n* = 3301)^21^ for the exposure, and the UK Biobank GWAS of RA (4412 cases, 365 085 controls)^22^ for the outcome, with protocols for serum protein measurements, case definitions, and genotyping defined elsewhere^21,22,24^.

### MR for causal inference

Two-sample MR^16^ was used to infer causal association, with protein level (using *cis*-pQTL variants as IVs) as the exposure, and RA (or RF) as the outcome. For the exposure, 40 (of 71) proteins with *cis*-pQTLs shared by the outcome GWAS were used^12^. For each outcome, summary statistics were obtained from the corresponding GWAS^13,14,16^.

MR requires three assumptions to be fulfilled (Fig. S1). First, the genetic variants should be associated with the exposure. Second, the genetic variants should not be associated with a confounder. Third, the genetic variants should be associated with the outcome only through the exposure; violation of this assumption is referred to as horizontal pleiotropy^9^. The first assumption was fulfilled by utilizing *cis*-pQTLs that reflect association with the exposure (plasma protein levels). The second and third assumptions were tested using sensitivity analyses including horizontal pleiotropy analyses that utilize the intercept term in MR Egger regression as an indicator, leave-one-out analyses to determine if a single SNP is driving the association and colocalization analyses as described below^25,26^.

Pruned *cis*-pQTL variants with linkage disequilibrium (LD) r^2^ < 0.01 for each protein were used as IVs to minimize the chances of a single nucleotide polymorphism (SNP) in LD being a confounder for the MR analysis. For proteins with only one independent pQTL variant after LD pruning, causal effect was determined using the Wald test, i.e., a ratio of effect per risk allele on RA to effect per risk allele on inverse-rank normalized protein levels. When multiple non-redundant pQTL variants were present, multi-SNP MR was conducted using fixed-effect inverse-variance weighted estimates. All MR analyses were conducted using the TwoSampleMR package in R^16^.

### Colocalization analysis

Colocalization analysis was conducted as an additional characterization of the inferred causal association in which shared genetic loci were identified between an exposure and an outcome^26^. The loci of proteins that were identified as putatively causal from the MR analyses were tested for colocalization with the loci of the outcomes to further explore the MR result and to consider potential confounders.

We first identified sentinel *cis*- and *trans*-pQTL variants for each protein. A locus was defined as within 1 Mb upstream or downstream (total span of 2 Mb) of each sentinel SNP. We then identified the SNPs within each locus that overlapped with the RA GWAS at *P* < 5.13 × 10^–9^ (0.05/9,739,304 variants)^13^. To estimate the probability that the overlapping locus reflects the same sentinel variant for both the protein and RA, we conducted a Bayesian test for colocalization of all SNPs in each locus using the *coloc* package in R. This method requires specifying a prior probability for a SNP being associated with RA only (p_1_), protein levels only (p_2_), and with both traits (p_12_). We applied the default values for p_1_ and p_2_ of 1 × 10^–4^ and p_12_ was specified as 1 × 10^–6^. We prioritized the analysis of the posterior probability (PP) of hypothesis H4, where one shared SNP is associated with both trait 1 and 2. Significant colocalization was defined as H4 > 0.90^27^.

## Results

**Figure 2:**
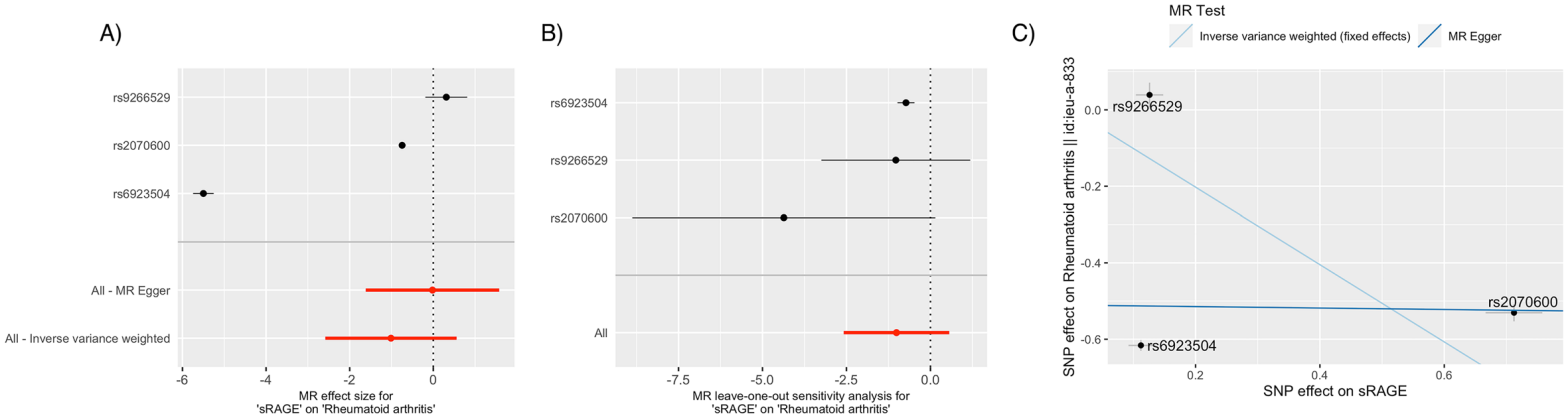
Mendelian randomization sensitivity analysis of sRAGE in relation to rheumatoid arthritis. (**A**): Forest plot of individual sRAGE cis-pQTL variant’s effect size (odds ratio per 1-standard deviation increment in inverse-rank normalized sRAGE level) in relation to RA. The fixed-effect inverse-variance weighted average effect of the SNPs were highly influenced by both rs2070600 and rs6923504 compared to rs9266529. (**B**): Leave-one-out analysis. Leaving rs2070600 out affected the confidence interval of the overall MR the most, while leaving rs6923504 did not affect the significance of the results. (**C**): Scatter plot of SNP effect size (odds ratio) in exposure GWAS (x-axis) and SNP effect size (standard deviation increment in inverse-rank normalized sRAGE level) in outcome GWAS (y-axis) for each cis-pQTL. rs2070600 is the driver of the causal association between sRAGE and RA. While rs2070600 is inversely associated with circulating sRAGE levels and positively associated with RA in their respective GWAS, note that the plotting algorithm transforms (x,y) to (− x,− y) if the x value is negative for plotting purposes (the slope and y-intercept remain the same).

**Figure 3:**
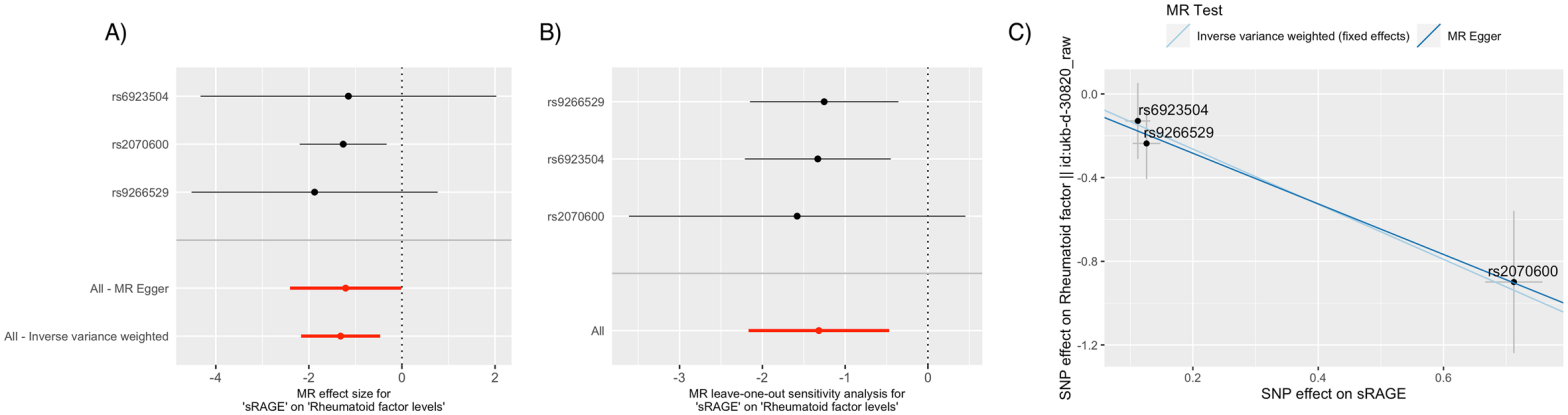
Mendelian randomization sensitivity analysis of sRAGE in relation to rheumatoid factor levels. (**A**): Forest plot of individual sRAGE cis-pQTL variant’s effect size (odds ratio per 1-standard deviation increment in inverse-rank normalized sRAGE level) in relation to plasma RF levels. rs2070600 had the narrowest confidence interval and contributed the most to the fixed-effect inverse-variance weighted effect of sRAGE on the outcome. (**B**): Leave-one-out analysis. Leaving rs2070600 out affected the confidence interval of the overall MR the most. (**C**): Scatter plot shows rs2070600 having the strongest contribution to the putatively causal association.

Table 1 summarizes MR results for two proteins (*P* < 0.05), and Table S1 presents the comprehensive MR results for all 40 proteins. Statistical significance was defined as *P* < 0.00125 (0.05/40). sRAGE was causally implicated (odds ratio [OR] per 1 standard deviation [SD] increment in inverse rank-normalized sRAGE levels = 0.364; 95% confidence interval [CI] 0.342–0.385; *P* = 6.40 × 10^–241^) with a protective effect (OR/ΔSD < 1) on RA with the sentinel *cis*-variant rs2070600 having the most replicable effect on the causal relationship passing the sensitivity analyses (Figs. 2, 3).

**Table 1:** Mendelian randomization results for rheumatoid arthritis (*p* < 0.05) and the corresponding Mendelian randomization for rheumatoid factor.

A.								
	Rheumatoid arthritis				Rheumatoid factor			
Exposure	n_snp_	Effect size	95% CI	P value	n_snp_	Effect size	SE	*P* value
sRAGE	n_snp_	0.364	[0.342; 0.385]	6.40E-241*	3	-1.318	0.43	0.02^†^
sICAM1	3	1.200	[1.026; 1.403]	0.02^†^	1	1.235	0.96	0.198

B.								
		Rheumatoid arthritis			Rheumatoid factor			
Exposure	SNP	Effect size	95% CI	P value	Effect size	SE	P value	
sRAGE	rs2070600 (6: 32151443T < C)	0.475	[0.446; 0.505 ]	5.25E–122*	− 1.262	0.48	0.008^†^	
	rs9266529 (6: 31342029A < G)	1.365	[0.829; 2.249]	0.222	− 1.877	1.35	0.164	
	rs6923504 (6: 32428186G < C)	0.004	[0.003; 0.005 ]	< E308*	− 1.150	1.62	0.478	
sICAM1	rs5498 (19:10395683G < A)	1.200	[1.026; 1.403 ]	0.02^†^	1.235	0.96	0.198	

*Denotes Bonferroni-corrected significance at *P* < 0.00125 (0.05/40).

^†^Denotes Bonferroni-corrected significance at *P* < 0.025 (0.05/2) for sRAGE and sICAM1.

Effect size for RA is odds ratio per 1 standard deviation [SD] increment in inverse rank-normalized sRAGE levels.

Effect size for RF is change in RF level (IU/mL) per 1 standard deviation [SD] increment in inverse rank-normalized sRAGE levels.

Single-SNP MR was performed using rs2070600 to further characterize the effect of rs2070600, which demonstrated a causal effect of sRAGE on RA (Wald test OR = 0.474; 95% CI 0.446–0.505; *P* = 5.25 × 10^–122^). While rs6923504 also contributed to the protective effect of sRAGE with a statistically significant *p* value, its effect size on RA was only 0.004, and its effect on RF was not statistically significant.sRAGE also was significant in MR analysis of RF levels as a secondary outcome (β, RF level change per sRAGE increment = − 1.318; SE = 0.434; *P* = 0.002). rs2070600 had the most substantial contribution to the RF causal relationship (Fig. 3), and single-SNP MR using rs2070600 revealed evidence of causality (Wald test β = − 1.263; SE = 0.477; *P* = 0.008).

Horizontal pleiotropy sensitivity analysis using MR Egger intercepts showed no significant horizontal pleiotropy between sRAGE and RA (*P*_pleiotropy_ = 0.336) or RF (*P*_pleiotropy_ = 0.843). Leave-one-out sensitivity analysis revealed that leaving rs2070600 out affected the confidence interval of the overall MR association more than any other pQTL SNPs for sRAGE.

In MR of both RA and RF, sRAGE was the only protein biomarker that passed the multiple testing corrected significance threshold. sRAGE was putatively casual and protective (OR/ΔSD < 1; Beta/ΔSD < 0) in relation to RA and RF.

The putatively causal protective relation of sRAGE to RA and RF was externally replicated, using the INTERVAL pQTL (rs2070600) for sRAGE as the exposure and the UK Biobank GWAS of RA as the outcome (Table 2; Fig. S2). The net protective effect of sRAGE was recapitulated both using the original FHS pQTL as the exposure and UK Biobank of RA as the outcome, as well as using the INTERVAL pQTL as the exposure and original MRC-IEU GWAS of RA was the outcome. The net inverse causal relation of sRAGE levels to RF levels was replicated with the INTERVAL pQTL.

**Table 2:** Mendelian randomization external replication for sRAGE in relation to rheumatoid arthritis and rheumatoid factor.

A.					
Exposure	Outcome	n_snp_/SNP	Effect size	95% CI	*P* value
FHS pQTL for sRAGE*	MRCIEU RA GWAS*	3	0.364	[0.342; 0.385]	6.40E−241
		rs2070600	0.475	[0.446; 0.505]	5.25E−122
		rs9266529	1.365	[0.829; 2.249]	0.222
		rs6923504	0.004	[0.003; 0.005]	< E308
	UK Biobank RA GWAS	3	0.420	[0.191; 0.924]	0.031
		rs2070600	0.475	[0.419; 0.539]	1.03E−30
		rs9266529	0.607	[0.431; 0.854]	0.004
		rs6923504	0.053	[0.034; 0.081]	8.77E−40
INTERVAL pQTL for sRAGE	MRCIEU RA GWAS*	1	0.394	[0.317; 0.472]	5.25E−122
		rs2070600			
	UK Biobank RA GWAS	1	0.395	[0.236; 0.553]	1.03E−30
		rs2070600			

B.					
Exposure	Outcome	n_snp_/SNP	Effect size	SE	*P* value
FHS pQTL for sRAGE*	UK Biobank RF GWAS*	3	− 1.318	0.43	0.002
		rs2070600	− 1.262	0.48	0.008
		rs9266529	− 1.877	1.35	0.164
		rs9266529	− 1.150	1.62	0.478
INTERVAL 1 pQTL for sRAGE	UK Biobank RF GWAS*	1	− 1.58	0.60	0.008
		rs2070600			

Effect size for RA is odds ratio per 1 standard deviation [SD] increment in inverse rank-normalized sRAGE levels.

Effect size for RF is the change in RF level per 1 standard deviation [SD] increment in inverse rank-normalized sRAGE levels.

* = dataset used for primary analysis.

External MR replication using the INTERVAL pQTL (*n* = 3301)^21^ and a smaller RA GWAS from the UK Biobank (4412 cases, 365 085 controls)^22^ was consistent with the primary finding. MR recapitulated the net protective effect of sRAGE. Replication with INTERVAL for the UK Biobank RF GWAS was also consistent with the primary MR analysis.

The minor T allele for rs2070600 was associated with 20–50% lower sRAGE levels in FHS participants (Table S2). This minor allele was associated with increased risk of RA and higher RF levels in the corresponding GWAS^13,16^ (OR [per risk allele] = 1.700; 95% CI 1.626–1.777; *P* = 3.60 × 10^–127^, and β [RF change per risk allele] = 0.899; SE = 0.340; *P* = 0.008, respectively).

Various other clinical characteristics such as mean age, percent women, body mass index, smoking status, history of diabetes, history of cardiovascular disease, and mean IL6 and CRP levels were also investigated by sRAGE levels. Quartile tabulation by sRAGE levels showed a significant association between sRAGE levels and BMI, current smoking status, and mean CRP levels (Tabe S3A). Cross-sectional multivariable regression model between sRAGE levels and clinical traits (history of diabetes and cardiovascular diseases) and inflammatory biomarker levels (Il-6 and CRP) revealed a significant inverse association between circulating sRAGE levels and CRP levels (Table S3B).

Colocalization analysis for sRAGE was conducted for three sentinel loci: rs4253272 (*trans*, Chromosome 4), rs116653040 (*trans* determined to be long-range *cis,* Chromosome 6), and rs2070600 (*cis*, Chromosome 6). At the posterior probability of > 0.90, only the rs116653040 locus (1 Mb window) significantly colocalized, reflecting an association of sRAGE levels with RA (PP.H4 = 1.00; Table S4). While the rs2070600 locus did not significantly colocalize (PP.H4 = 5.08 × 10^–48^), rs116653040 is in significant linkage disequilibrium with rs2070600 (R^2^ = 0.3041, *P* < 0.0001), indicating that rs116653040 is associated with sRAGE level and RA while acting as a long-range *cis-*locus for sRAGE. Thus, the causal association of sRAGE with RA from MR is strongly driven by rs2070600, although it may confer effects in conjunction with other SNPs in LD (e.g. with rs9266529 [the other *cis*-pQTL used as an IV for sRAGE]; r^2^ = 0.0438, *P* = 0.0032)^28^ that modulate sRAGE levels.

## Discussion

**Figure 4:**
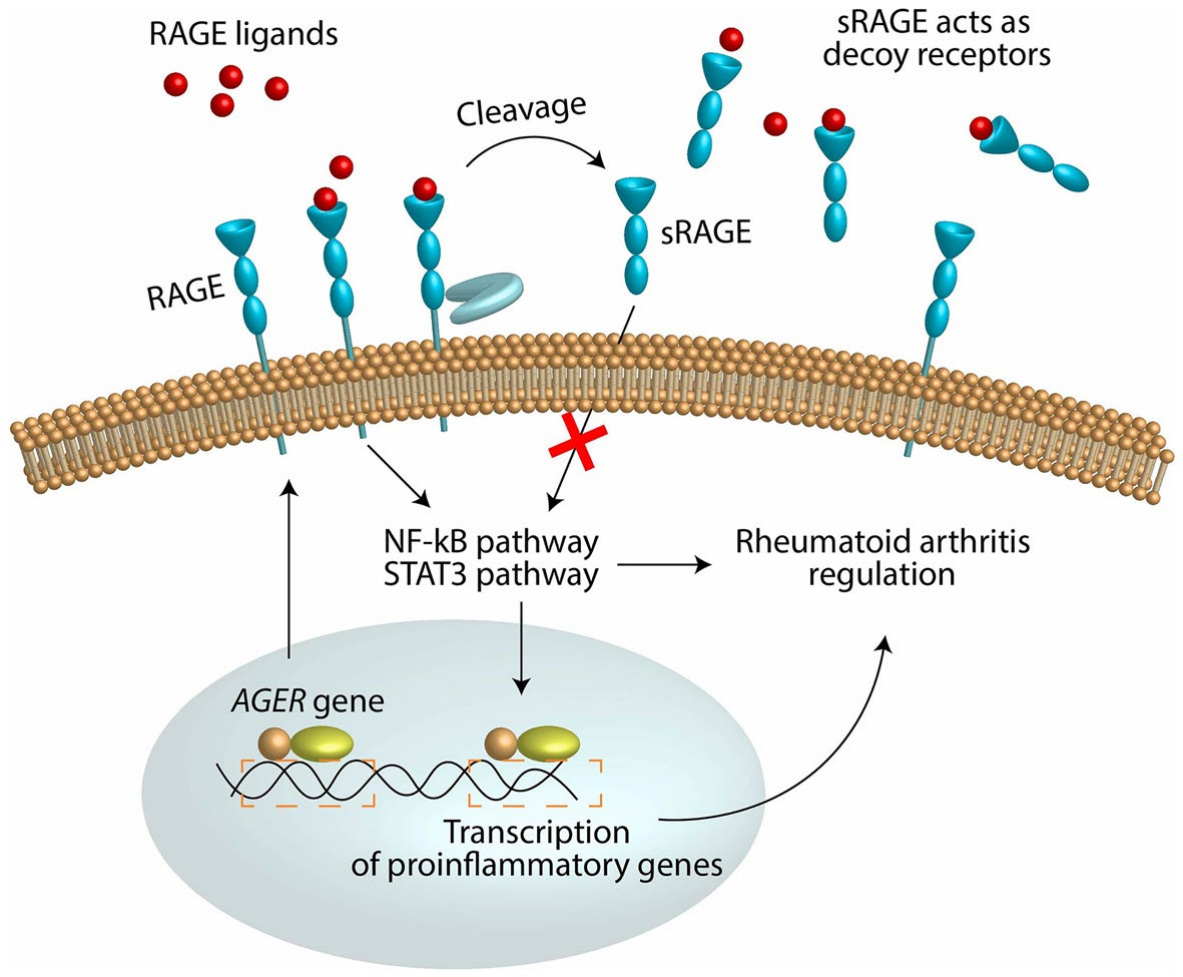
The protective role of sRAGE in relation to rheumatoid arthritis. Depiction of the protective mechanism of sRAGE in relation to RA. RAGE is a membrane-bound receptor that triggers pro-inflammatory pathways implicated with RA. sRAGE, a circulating form of RAGE, acts as a decoy receptor for RAGE ligands and therefore downregulates pro-inflammatory pathways.

Using an integrative genomic strategy, we identified sRAGE as putatively causal and protective protein against both RA and RF. sRAGE is a soluble form of RAGE, a transmembrane protein coded by the *AGER* gene (Fig. 4). *AGER* is located in the human leukocyte antigen (HLA) class III locus, near *HLA-DRB1* and the HLA class II locus, both of which have been reported to be associated with RA^2^. Ligands that bind to membrane-bound RAGE, including advanced glycation end products, S100 proteins, and high mobility group box-1 protein (HMGB1), trigger proinflammatory pathways^29^. Circulating sRAGE is derived from proteolytic cleavage of membrane-bound RAGE (mRAGE) or via endogenous secretion of an alternatively spliced isoform (esRAGE) that lacks the trans-membrane domain of the RAGE protein. sRAGE acts as a decoy receptor and binds to RAGE ligands without inciting RAGE-mediated inflammatory signalling, explaining its protective effect. Indeed, a recent study^30^ found that sRAGE-overexpressing mesenchymal stem cells (MSCs) had reduced proinflammatory molecule production and increased immunomodulatory molecule expression. Similarly, IL-1Ra-knockout mice transplanted with sRAGE-overproducing MSCs demonstrated a reduction in inflammatory arthritis^30^. Of note, methotrexate, a first-line RA treatment, acts in part by directly binding to the RAGE ligand HMGB1 to inhibit the HMGB1/RAGE pathway^31^.

Of the genetic variants driving the observed causal effect of sRAGE, we decided to focus our analysis on rs2070600 based on the sensitivity analysis ruling rs6923504 out as a significant contribution to the association. rs2070600 is a missense variant in *AGER* exon 3 with higher prevalence in RA patients^29^. The amino acid substitution (Gly82Ser) resides at the ligand-binding domain and increases the affinity for RAGE ligands^29^, enhancing proinflammatory signalling. This polymorphism is thought to simultaneously make RAGE less susceptible to cell surface RAGE cleavage^29^, reducing the generation of sRAGE. This has the dual effects of increasing RAGE proinflammatory ligand-binding and decreasing availability of sRAGE to act as a decoy receptor for ligands. Consistent with these joint effects, we found that the rs2070600 Ser (versus Gly) substitution was associated with lower circulating sRAGE levels in FHS participants^12^ and positively associated with both RA and RF levels in GWAS^13,16^.

Previous proteomic MR studies of RA have reported IL-6^32^, CRP^33^, and sex hormone-binding globulin^34^ as causal biomarkers of RA. While IL-6 and sex hormone-binding globulin were not in our panel of proteins, we found that CRP was not causal for RA (*P* = 0.512; Table S1). We posit that the 22 SNPs used for CRP as the exposure in the prior MR study did not distinguish *cis-* or *trans-* pQTLs and spanned multiple chromosomes—and therefore may have contributed to horizontal pleiotropy from other genes remote from the CRP locus.

The *AGER* gene is located in the human leukocyte antigen (HLA) class III locus on chromosome 6, between the HLA class I and the HLA class II locus, which has been reported to be associated with RA^2^. Colocalization analysis hinted at the presence of other long-range effects on sRAGE and RA in addition to the modulation by missense variant rs2070600. Since the HLA locus is highly polymorphic and with sizable LD across the region^35–37^, the relationship at the HLA class II locus (e.g. rs116653040) was interrogated.

A query of National Cancer Institute's LDtrait Tool^38^ for variants in LD with rs2070600 or rs116653040 that have been reported to be associated with RA in Europeans revealed a signal for rs6910071 (OR [95% CI] = 2.73–3.03, *P* = 1 × 10^–299^ from a GWAS with 5,539 cases and 20,169 controls)^39^. rs6910071 is a tag SNP for the HLA-DRB1*0401 allele near *C6orf10*^40^. rs6910071 is in LD with rs2070600 with r^2^ = 0.1081 (*P* < 0.0001).

Based on reported whole blood expression quantitative loci (eQTL) in GTEx^41^, rs2070600 was found to be significantly associated with expression of *HLA-DQA2* (*p* = 1.1 × 10^–13^) and *HLA-DRB1* (*P* = 2.2 × 10^–5^). rs116653040, the *trans-*pQTL for sRAGE in LD with rs2070600 (r^2^ = 0.3041, *P* < 0.0001), was associated with *HLA-C* in whole blood (*p* = 4.9E−11). rs6910071, the RA-associated *HLA-DRB1* tag-SNP in LD with rs2070600, was associated with *HLA-DQA2* (*P* = 2.4 × 10^–21^) and *HLA-DRB1* (*P* = 1.5 × 10^–7^). All three genes, *HLA-DQA2*, *HLA-DRB1*, and *HLA-C*, have been reported to be associated with RA^2,42^. The degree to which the causal association of sRAGE with RA is driven by the RAGE-mediated inflammatory pathways, by interaction with HLA class II genes, or both, warrants further investigation.

Additionally, rs2070600 is associated with other phenotypes including asthma^43^, lung function^44^, and celiac disease^45^. While the diverse role of missense variant rs2070600 raises the spectre of horizontal pleiotropy, we posit that the effects are likely explained by sRAGE and its effect on inflammatory signalling. Therefore, another consideration of this missense variant is vertical pleiotropy, whereby the additional traits associated with rs2070600 represent the downstream effects of the exposure and do not violate MR assumptions and premises^25,46^.

Our study has several limitations. First, while we utilized the FHS pQTL variants identified from 71 CVD-related proteins, they are not representative of the entire human plasma proteome. While we rationalize the use of the FHS pQTLs associated with CVD-related proteins due to the purported link between CVD and RA, a similar analysis with more comprehensive pQTLs could reveal additional significant pathways and potential confounders. There remains a need for a pan-protein pQTL resource with sufficient sample size and SNP associations to run reliable Mendelian randomization analyses. The FHS pQTL dataset based on GWAS of nearly 7000 individuals allowed us to conduct a statistically powerful MR with a sufficient number of SNPs. Second, the proteins were measured in plasma, which may yield conclusions not translatable to tissue-specific protein effects. While circulating sRAGE levels are correlated with synovial fluid sRAGE levels (r_s_ = 0.48, *P* = 0.0002)^47^, our findings should be confirmed in tissue-specific settings. Third, since the SNPs accounting for the causal association between sRAGE and RA are in close proximity with HLA class II, the interaction between RAGE-mediated effects on RA with HLA genes should be delineated further. Fourth, while MR testing allowed inference of causal effects of protein levels on RA and RF, further cell and animal studies are warranted. If our findings are confirmed, modulation of AGER/RAGE to reduce inflammatory signalling, by altering sRAGE production, may lead to novel therapies for RA.

## Conclusions

Through Mendelian randomization using pQTLs of CVD-related proteins along with GWAS of RA and RF, sRAGE was identified as putatively protective for both RA and RF levels. Given that sRAGE was previously identified as a potential inhibitor of RAGE-mediated inflammation related to RA, we hypothesize that the *AGER*/RAGE axis is a promising therapeutic target for RA.

## Supplementary Information

Supplementary Information.

## Data Availability

The study utilized de-identified human data consisting of summary statistics of genome-wide association studies and de-identified aggregate clinical data from the Framingham Heart Study. The datasets generated and/or analysed during the current study are available in the dbGaP and BioLINCC repositories. The data used for this study are all publicly available^12–14,16,21,22^. The FHS pQTL resource can be found at https://www.nature.com/articles/s41467-018-05512-x^12^ and the INTERVAL pQTL can be found at https://www.ncbi.nlm.nih.gov/pmc/articles/PMC6093935/^21^. The RA GWAS from Okada et al. accessed via MRCIEU can be found at https://gwas.mrcieu.ac.uk/datasets/ieu-a-833/^13,16^, the UK Biobank GWAS of RA can be found at http://pheweb.sph.umich.edu/SAIGE-UKB/pheno/714.1^22,24^, and the UK Biobank GWAS of RF can be found at https://gwas.mrcieu.ac.uk/datasets/ukb-d-30820_raw/^16,48^. The results of the Mendelian randomization analysis from this study are available in full in Supplementary Table S1.
