# Supplementary material for: Integrative Mendelian randomization reveals the soluble receptor for advanced glycation end products as protective in relation to rheumatoid arthritis
Source: Sci Rep. 2023 May 17;13:8002. doi: 10.1038/s41598-023-35098-4 (PMC10192300; doi:10.1038/s41598-023-35098-4)
Supplement: Supplementary file 1 — Supplementary Information. [file 41598_2023_35098_MOESM1_ESM.docx]

**Supplementary Material**


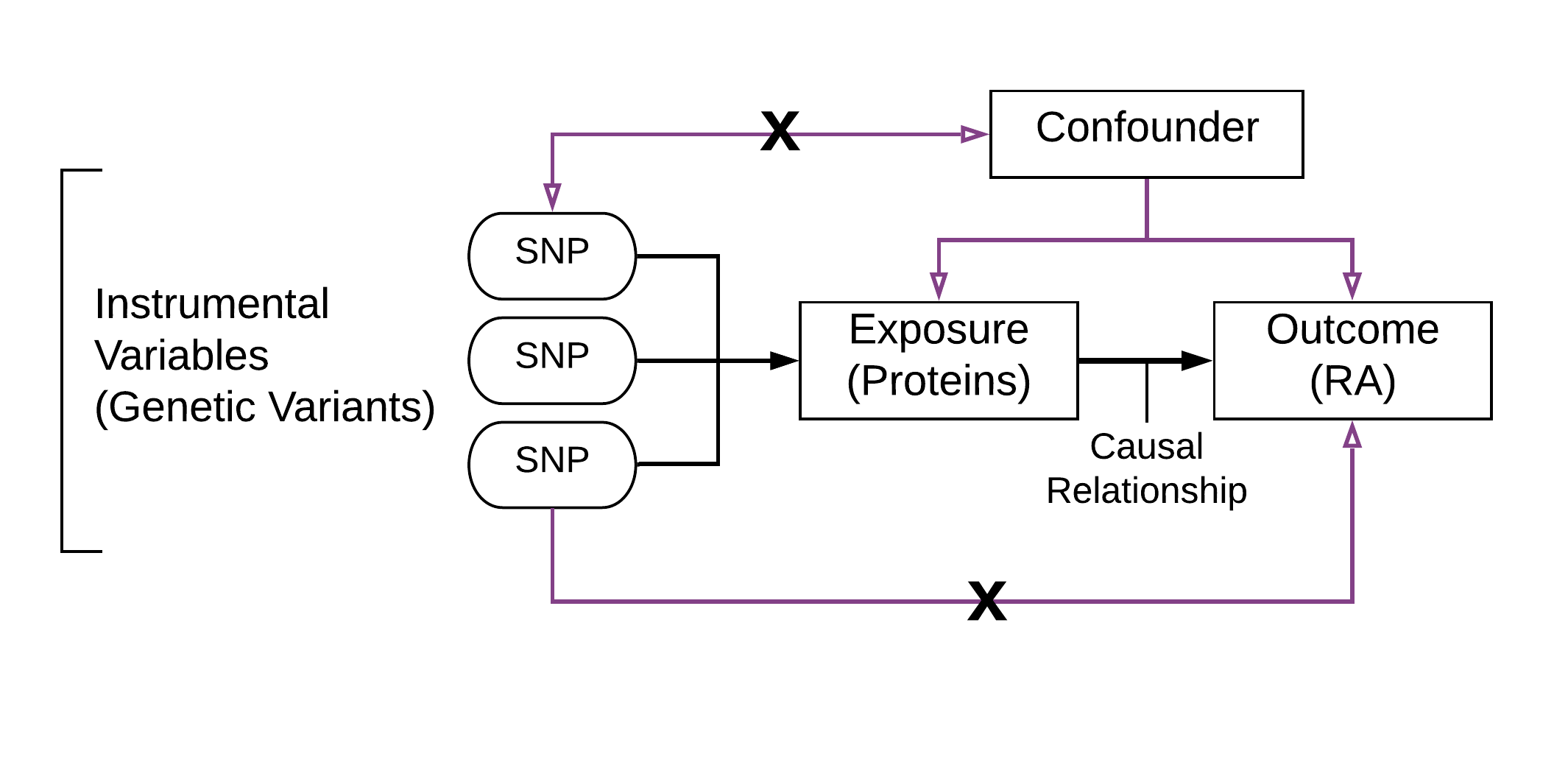


**Supplementary Figure S1. Mendelian Randomization Overview**

Overview of Mendelian randomization, which can be used to infer causal relationship between an exposure (e.g. protein level) and an outcome (e.g. RA and RF). MR assumes that instrumental variables (IVs) are associated with the exposure, influences the outcome via the exposure only, and are not associated with confounders.

**Supplementary Table S1. Mendelian Randomization Results for 40 Proteins in Relation to Rheumatoid Arthritis**

| **Protein** | **n_snp_** | **OR** | **95% CI** | **P Value** |
| --- | --- | --- | --- | --- |
| **sRAGE** | 3 | 0.364 | 0.342-0.385 | 6.40E-241* |
| **sICAM1** | 1 | 1.199 | 1.029-1.397 | 0.020 |
| **NTproBNP** | 1 | 1.121 | 0.979-1.283 | 0.096 |
| **MMP9** | 1 | 1.124 | 0.97-1.301 | 0.119 |
| **LPA** | 11 | 0.969 | 0.926-1.012 | 0.163 |
| **EFEMP1** | 2 | 0.914 | 0.802-1.039 | 0.170 |
| **CD14** | 1 | 0.941 | 0.858-1.031 | 0.196 |
| **GMP140** | 3 | 1.050 | 0.967-1.138 | 0.242 |
| **CD40L** | 1 | 1.254 | 0.853-1.843 | 0.249 |
| **MMP8** | 1 | 0.863 | 0.666-1.116 | 0.262 |
| **ADM** | 2 | 0.909 | 0.759-1.088 | 0.299 |
| **C2** | 2 | 0.949 | 0.855-1.052 | 0.320 |
| **BCHE** | 1 | 0.959 | 0.881-1.042 | 0.323 |
| **CLEC3B** | 1 | 0.911 | 0.757-1.095 | 0.323 |
| **GDF15** | 1 | 1.040 | 0.96-1.125 | 0.332 |
| **NCAM** | 3 | 0.972 | 0.906-1.043 | 0.433 |
| **CRP** | 1 | 0.935 | 0.766-1.141 | 0.512 |
| **COL18A1** | 1 | 0.942 | 0.787-1.126 | 0.512 |
| **DPP4** | 1 | 1.101 | 0.825-1.469 | 0.512 |
| **CD5L** | 1 | 0.965 | 0.867-1.073 | 0.516 |
| **FGG** | 1 | 1.061 | 0.887-1.269 | 0.516 |
| **MCAM** | 1 | 1.072 | 0.868-1.323 | 0.516 |
| **GP5** | 1 | 1.060 | 0.866-1.296 | 0.572 |
| **Cystatin C** | 1 | 1.026 | 0.938-1.12 | 0.576 |
| **Ceruloplasmin** | 1 | 0.963 | 0.841-1.1 | 0.576 |
| **KLKB1** | 2 | 1.028 | 0.932-1.134 | 0.578 |
| **UCMGP** | 1 | 1.039 | 0.908-1.187 | 0.579 |
| **Resistin** | 1 | 0.944 | 0.752-1.185 | 0.623 |
| **CXCL16** | 2 | 0.970 | 0.84-1.119 | 0.678 |
| **SAA1** | 1 | 1.018 | 0.931-1.111 | 0.697 |
| **MPO** | 2 | 1.024 | 0.894-1.172 | 0.732 |
| **CNTN1** | 2 | 1.018 | 0.905-1.143 | 0.771 |
| **PON1** | 3 | 0.996 | 0.965-1.027 | 0.816 |
| **SERPINA10** | 4 | 1.007 | 0.95-1.066 | 0.821 |
| **AGP1** | 1 | 0.972 | 0.749-1.259 | 0.827 |
| **A1M** | 1 | 1.035 | 0.761-1.406 | 0.827 |
| **REG1A** | 2 | 1.007 | 0.906-1.117 | 0.901 |
| **GRN** | 1 | 1.000 | 0.873-1.145 | 1.000 |
| **sGP130** | 1 | 1.000 | 0.834-1.198 | 1.000 |
| **HPX** | 1 | 1.000 | 0.853-1.172 | 1.000 |

* denotes Bonferroni-corrected significance at P<0.00125 (0.05/40).

MR results for all 40 proteins that were tested in MR analysis in relation to RA. sRAGE and sICAM1 were the only significant proteins, with sRAGE passing the Bonferroni-corrected significance threshold for multiple testing (p<0.05/34) and sICAM1 being only nominally significant (p<0.05). The OR for the MR results is the odds ratio per 1 SD increment in inverse rank-normalized protein levels.

**
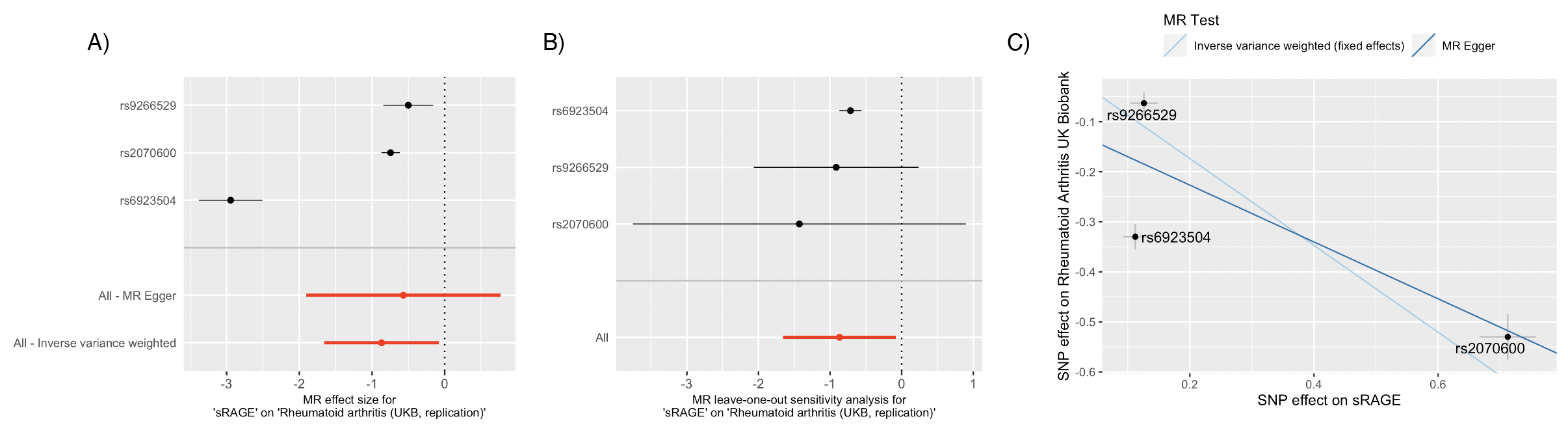
**

**Supplementary Figure S2. Replication Mendelian Randomization Sensitivity Analysis of sRAGE in Relation to Rheumatoid Arthritis (UK Biobank)**

(A): Forest plot of individual sRAGE cis-pQTL variant’s effect size in relation to the replication RA cohort. rs2070600 similarly had the narrowest confidence interval and contributed the most to the inverse-variance weighted effect of sRAGE on the outcome. (B): Leave-one-out analysis. Leaving rs2070600 out affected the confidence interval of the overall MR the most, secondary to rs9266529. (C): Scatter plot shows rs2070600 having the strongest contribution to the putatively causal association, similar to Fig 2 and Fig 3.

**Supplementary Table S2. sRAGE Levels in Framingham Heart Study Participants**

|  |  | sRAGE Levels (pg/mL) | | | | | | |
| --- | --- | --- | --- | --- | --- | --- | --- | --- |
| **rs2070600** | **N** | **Mean** | **Std** | **Minimum** | **25th** | **Median** | **75th** | **Maximum** |
| C/C | 6260 | 3633.920 | 1164 | 269 | 2810 | 3500 | 4310 | 12600 |
| C/T | 558 | 2895.400 | 948 | 615 | 2230 | 2810 | 3390 | 6380 |
| T/T | 13 | 1661.540 | 432 | 850 | 1450 | 1610 | 2000 | 2470 |

A distribution of plasma sRAGE level by the genotype of rs2070600 was generated. There were a total of 6831 FHS participants with information on rs2070600 genotype from the 1000G imputation and plasma protein measurements. Among these 6831 FHS participants, a total of 558 individuals carried one minor T allele, 13 were homozygotes of the T allele, and 6260 carried the major (C) allele.

**Table S3A. Association of Circulating sRAGE Concentration with Clinical Characteristics**

|  | **Quartile 1** | **Quartile 2** | **Quartile 3** | **Quartile 4** | **P value** |
| --- | --- | --- | --- | --- | --- |
| **N** | 1714 | 1689 | 1709 | 1698 |  |
| **sRAGE range (pg/mL)** | 269-2750 | 2760-3430 | 3440-4240 | 4250-12600 |  |
| **Mean Age (yrs)** | 51.21 | 49.60 | 48.26 | 48.42 |  |
| **Women (%)** | 43.64 | 49.56 | 54.77 | 64.72 |  |
| **Body Mass Index (kg/m^2^)** | 29.21 | 28.09 | 26.70 | 25.68 | <1E-17* |
| **Current smoker (%)** | 16.76 | 14.74 | 13.29 | 13.97 | 0.0006* |
| **Hx of diabetes (%)** | 8.82 | 7.64 | 5.62 | 3.95 | 0.122 |
| **Hx of cardiovascular disease (%)** | 16.74 | 13.44 | 12.4 | 11.78 | 0.978 |
| **Mean IL6 levels (pg/mL)** | 6.23 | 6.26 | 6.37 | 6.41 | 0.2 |
| **Mean CRP levels (mg/L)** | 2.77 | 2.01 | 1.91 | 1.52 | 1.10E-08* |

Clinical characteristics (mean age, percent females, body mass index, smoker status, history of diabetes, and history of cardiovascular disease) were tabulated by quartiles of plasma sRAGE concentrations. Trend p-values were age and sex adjusted.

**Table S3B.** **Association of Circulating sRAGE Concentration with Clinical Characteristics**

| **Trait** | **Beta** | **SE** | **P-value** |
| --- | --- | --- | --- |
| **Hx of diabetes** | -0.595 | 0.353 | 0.092 |
| **Hx of CVD** | 0.019 | 0.276 | 0.945 |
| **IL-6 levels** | 0.005 | 0.005 | 0.356 |
| **CRP levels** | -0.338 | 0.046 | 2.32E-12* |

Covariates: age, sex, BMI, study cohort, current smoker (yes/no), former smoker (yes/no)

We conducted multivariable regression models to test significant associations between circulating sRAGE levels (log-transformed) and history of diabetes, history of CVD, or circulating levels of inflammatory markers IL-6 and CRP (log-transformed).

**Supplementary Table S4.** **Colocalization Analysis of sRAGE in Relation to Rheumatoid Arthritis**

| **Protein** | **Sentinel SNP** | **Cis/Trans** | **Chr** | **Position** | **PP.H3** | **PP.H4** | **Causal SNP** |
| --- | --- | --- | --- | --- | --- | --- | --- |
| sRAGE | rs2070600 | Cis | 6 | 32151443 | **1.00E+00** | 5.08E-48 | rs9275184 |
|  | rs116653040 | Trans/Long-acting Cis | 6 | 31109567 | 2.79E-05 | **1.00E+00** | rs34562262 |
|  | rs4253272 | Trans | 4 | 187163614 | 3.72E-02 | 1.12E-02 | NA |

Three *cis-*pQTL variants for soluble receptor for advanced glycation end products (sRAGE) coincided with RA GWAS variants. Colocalization was conducted on three sentinel SNPs for sRAGE which included rs4253272 (*trans,* Chr 4), rs116653040 (*trans,* Chr 6), and rs2070600 (*cis ,* Chr 6). While only the *trans­*-locus surrounding rs116653040 colocalized with RA (PP.H4=1.000), rs116653040 is also in significant linkage disequilibrium with rs2070600 (R^2^=0.3041, p<0.0001).

(A)


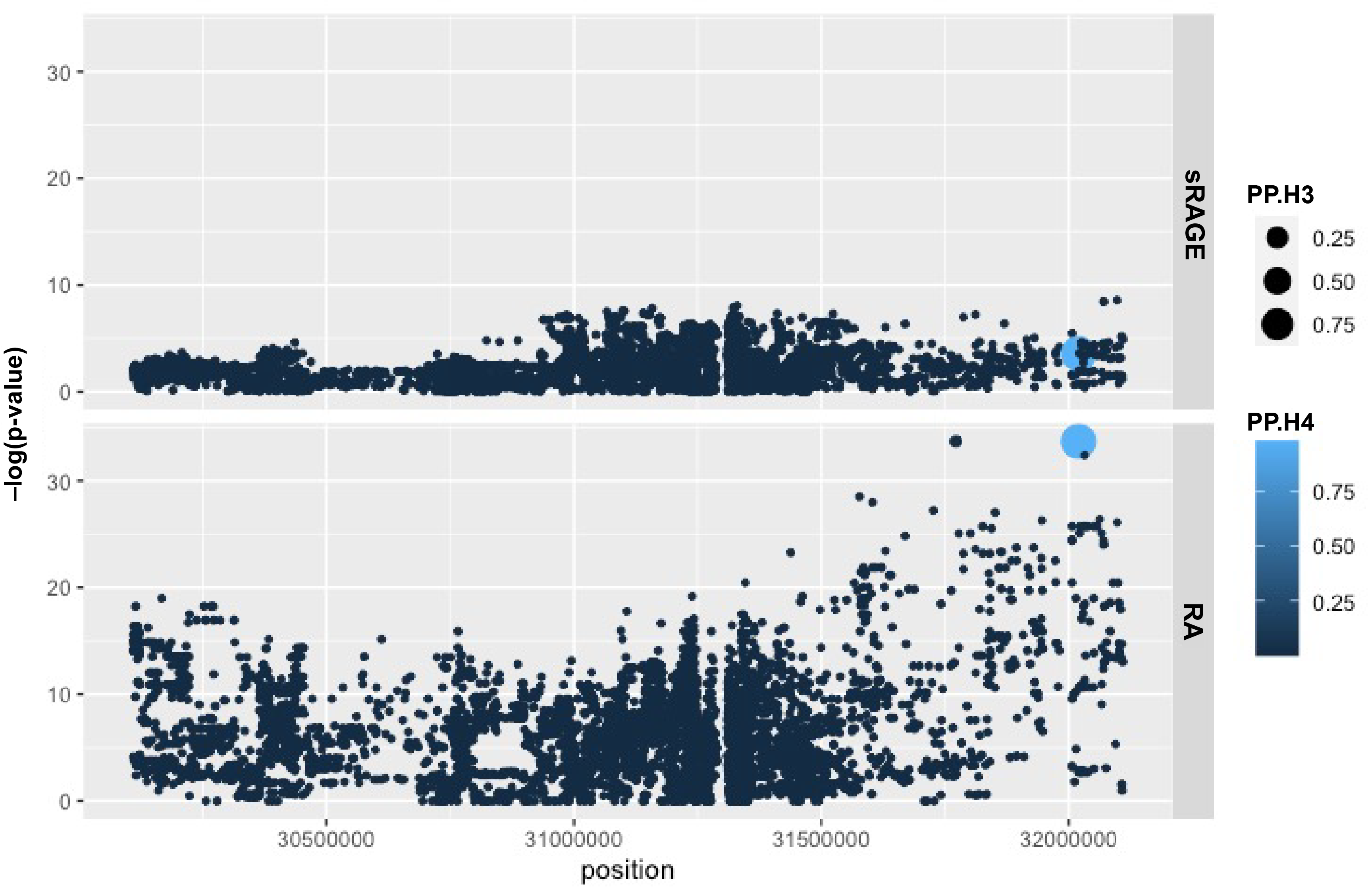


(B)


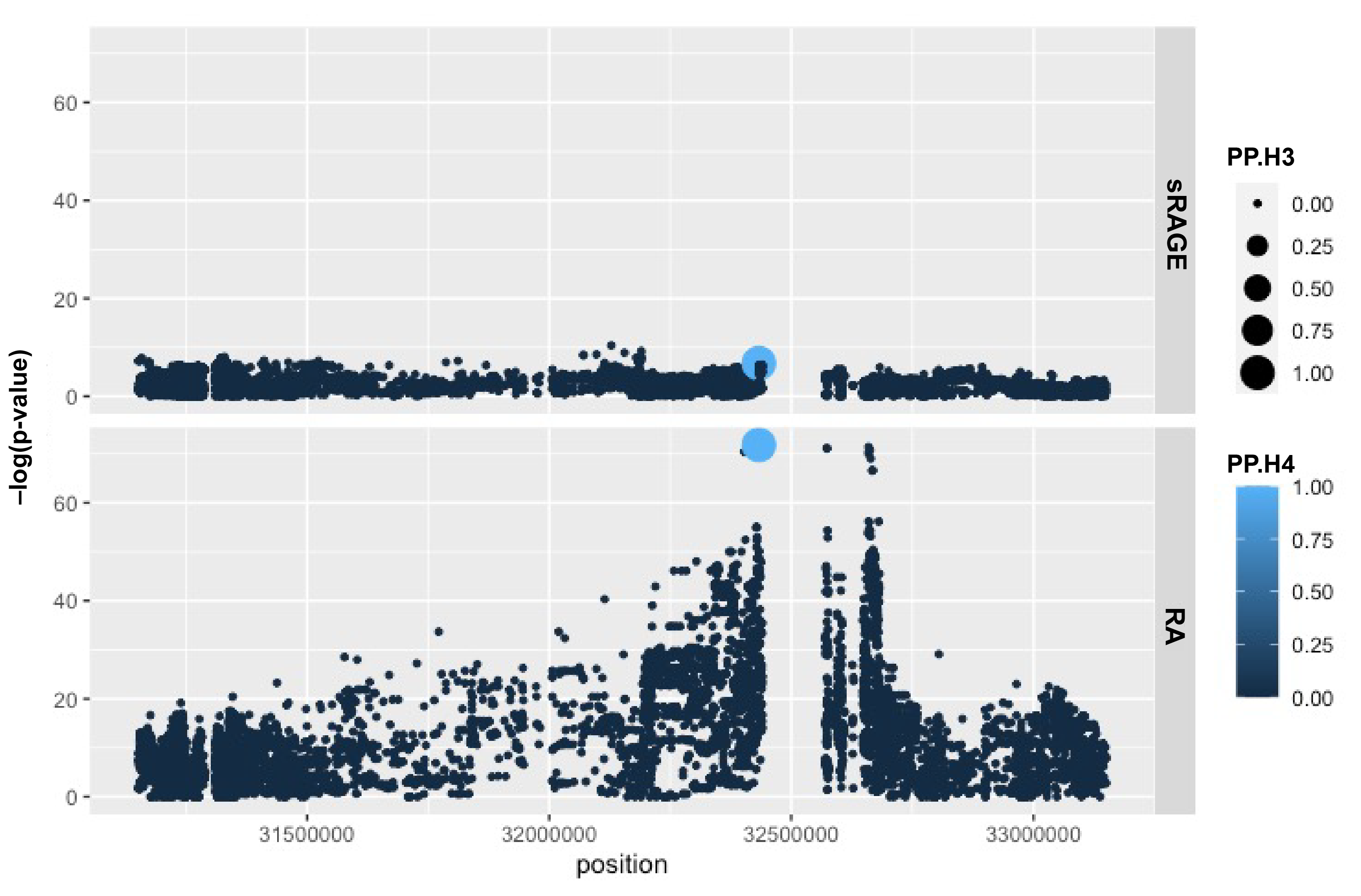


(C)


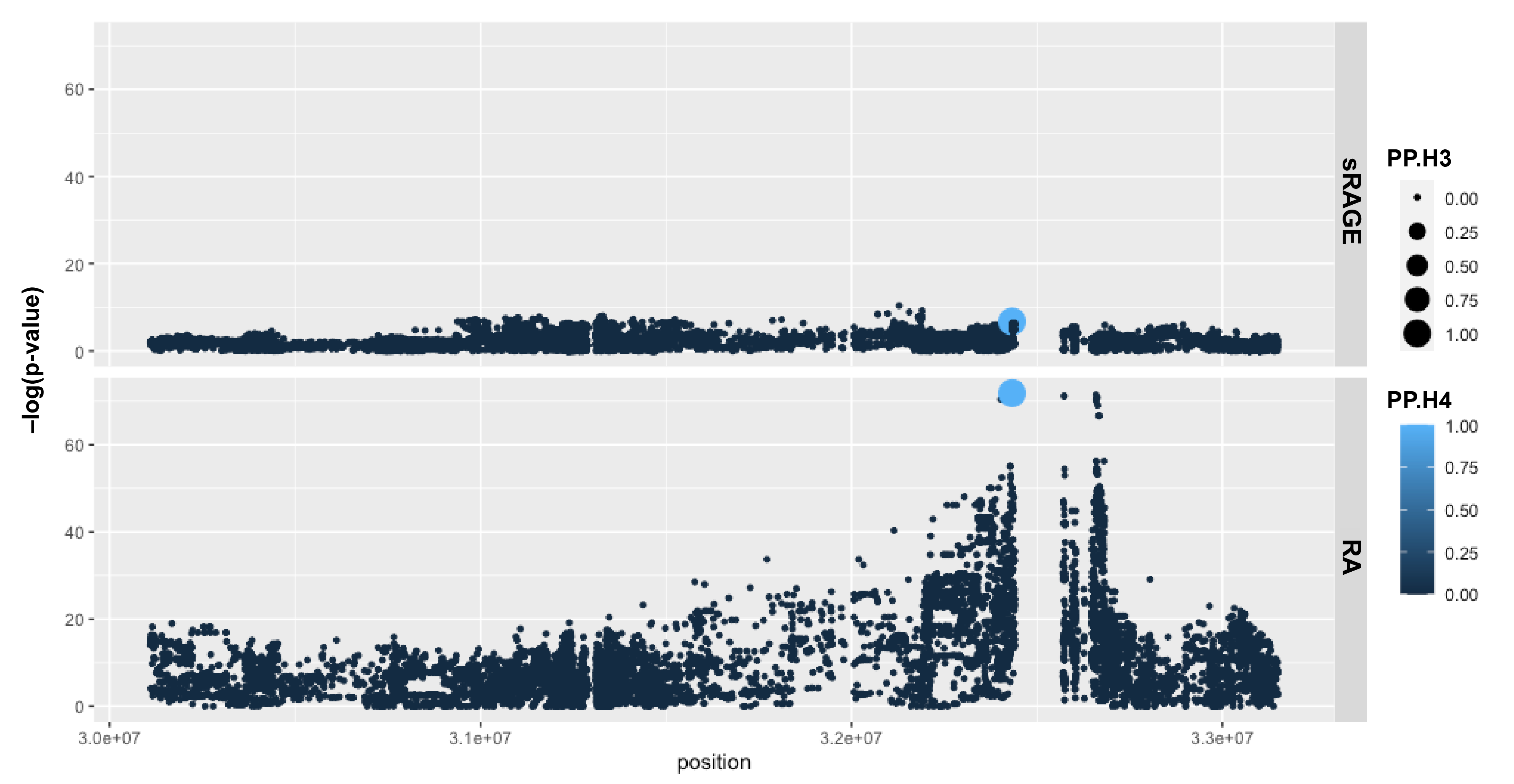


**Supplementary Figure S3. Colocalization Plot of Shared Variants Between sRAGE and RA**

(A): colocalization overlap of the rs116653040 region between 6:30109567-32109567. The top row refers to the pQTL and the bottom row refers to the RA GWAS. The y-axis refers to the negative log of the p-values from the GWAS. PP refers to posterior probability, with dots referring to H3 (both traits are associated, but with different causal variants) and color-scale referring to H4 (both traits are associated and share the same single causal variant). (B): colocalization rs2070600 region between 6:31151443-33151443. (C): combined colocalization of regions from 6:30109567-33151443 encompassing both the rs116653040 and rs2070600 regions.
